# Mechanism-Based Redesign of GAP to Activate Oncogenic Ras

**DOI:** 10.1021/jacs.3c04330

**Published:** 2023-09-08

**Authors:** Dénes Berta, Sascha Gehrke, Kinga Nyíri, Beáta G. Vértessy, Edina Rosta

**Affiliations:** † Department of Physics and Astronomy, University College London, Gower Street, London WC1E 6BT, United Kingdom; ‡ Institute of Enzymology, Research Centre for Natural Sciences, Magyar tudósok körútja 2, Budapest 1117, Hungary; § Department of Applied Biotechnology and Food Science, Budapest University of Technology and Economics, Budafoki út 6-8, Budapest 1111, Hungary

## Abstract

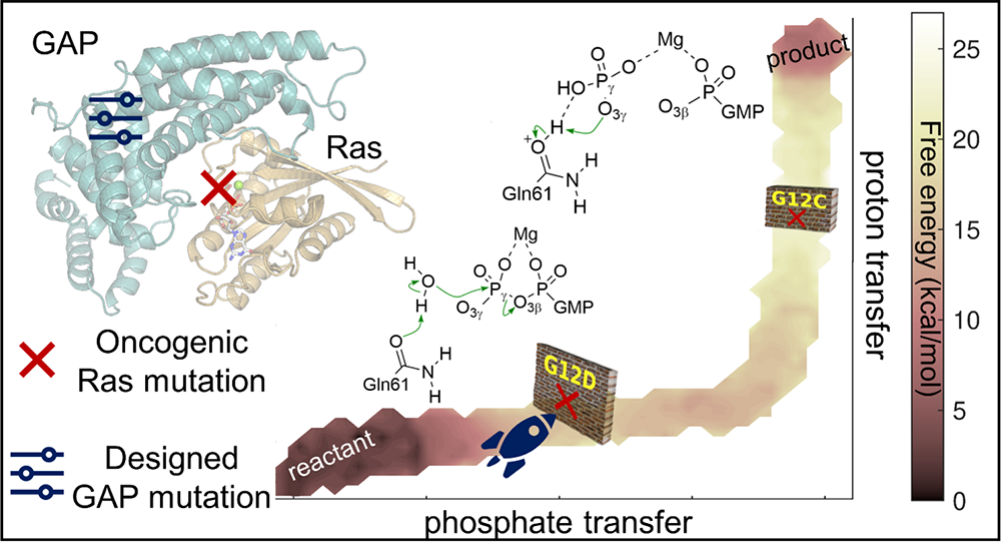

Ras GTPases play a crucial role in cell signaling pathways. Mutations of the Ras gene occur in about one third of cancerous cell lines and are often associated with detrimental clinical prognosis. Hot spot residues Gly12, Gly13, and Gln61 cover 97% of oncogenic mutations, which impair the enzymatic activity in Ras. Using QM/MM free energy calculations, we present a two-step mechanism for the GTP hydrolysis catalyzed by the wild-type Ras.GAP complex. We found that the deprotonation of the catalytic water takes place via the Gln61 as a transient Brønsted base. We also determined the reaction profiles for key oncogenic Ras mutants G12D and G12C using QM/MM minimizations, matching the experimentally observed loss of catalytic activity, thereby validating our reaction mechanism. Using the optimized reaction paths, we devised a fast and accurate procedure to design GAP mutants that activate G12D Ras. We replaced GAP residues near the active site and determined the activation barrier for 190 single mutants. We furthermore built a machine learning for ultrafast screening, by fast prediction of the barrier heights, tested both on the single and double mutations. This work demonstrates that fast and accurate screening can be accomplished via QM/MM reaction path optimizations to design protein sequences with increased catalytic activity. Several GAP mutations are predicted to re-enable catalysis in oncogenic G12D, offering a promising avenue to overcome aberrant Ras-driven signal transduction by activating enzymatic activity instead of inhibition. The outlined computational screening protocol is readily applicable for designing ligands and cofactors analogously.

## Introduction

The Ras protein isoforms are essential components of key signaling networks to promote cell proliferation and survival.^1^ Ras is the most frequently mutated enzyme in all cancer. Ras oncogenes are involved in more than 30% of all human cancer,^2−5^ including 98% of pancreatic cancer,^6^ 52% of colorectal cancer^7,8^ as well as in melanoma,^9−11^ and lung cancer.^12,13^ Additionally, the prognosis for Ras-positive cancer cases is significantly worse than without Ras mutations.^7,11,14−16^ Ras was previously called “undruggable”;^17−19^ it was only after three decades of extensive research that approved drugs reached the clinic targeting the G12C mutation specifically. New therapies, for more Ras mutations, are therefore highly sought after.^20^

**Figure 1:**
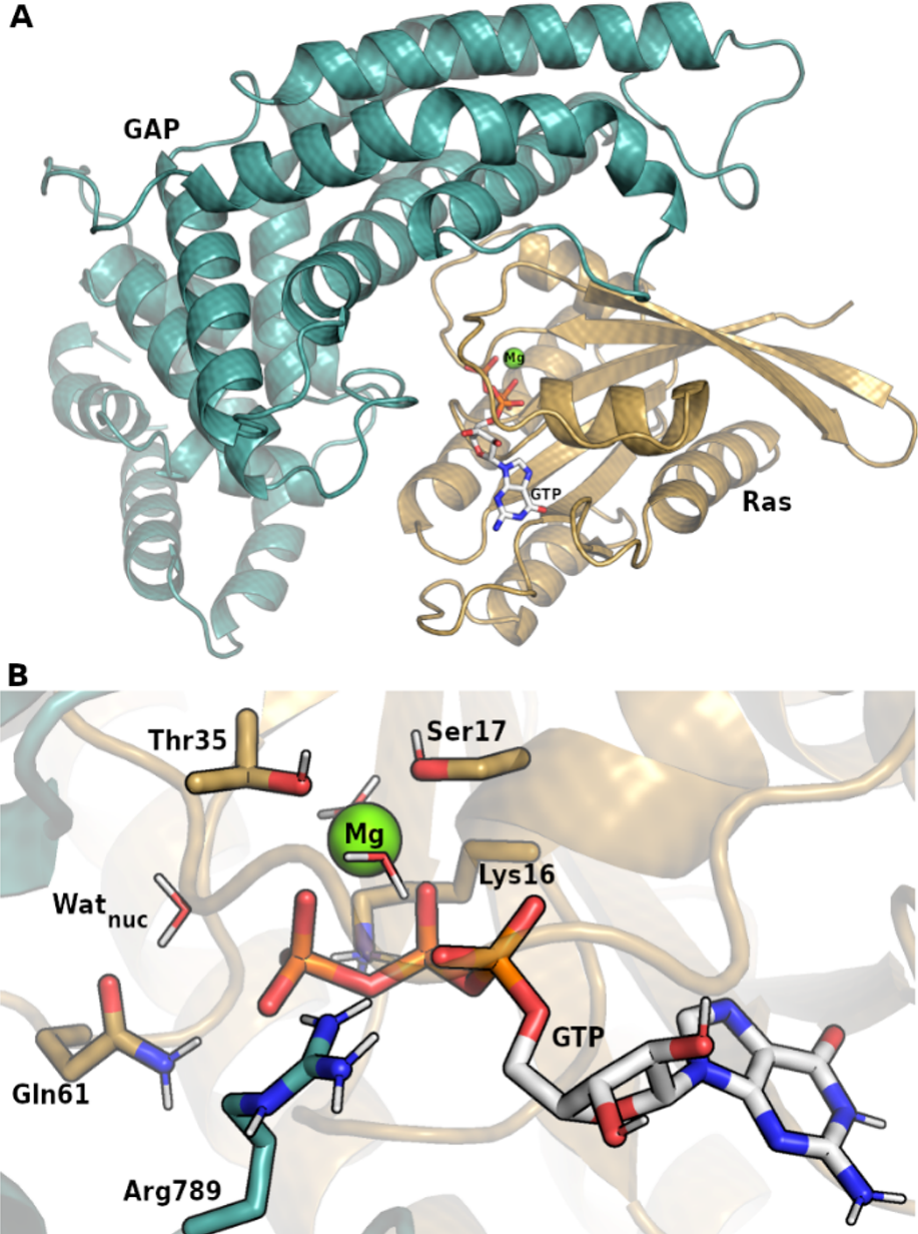
(A) Ras (gold cartoon)-GAP (blue cartoon) model based on PDB ID 1WQ1. (B) GTP (white sticks) alongside with Mg^2+^-coordinating residues. Arginine finger (blue sticks) from p120GAP coordinates the GTP.

Ras is a small GTPase that binds GTP with very high, picomolar affinity (Figure 1).^17^ In its GTP-bound form, Ras is active and promotes signaling for cell proliferation.^21^ To turn signaling off,^22,23^ Ras hydrolyses GTP to GDP with the help of GTPase-activating proteins (GAPs), typically p120GAP or Ras p21.^24,25^ GAP completes the environment around the active site (Figure 1A); it contains key conserved motifs, including an arginine finger (Figure 1B)^26^ to enable effective catalysis. However, key oncogenic mutations render Ras catalytically inactive, and thus, Ras stays in its active signaling, GTP-bound form.^27^

In a recent experimental work, the RGS3 domain, which serves as GAP for other G proteins, was found to recover catalytic activity of G12C Ras compared with intrinsic or NF1-catalyzed hydrolysis.^28^ This validates an approach that targets oncogenic Ras by restoring its activity, instead of modulating the signaling by the inhibition of downstream effectors. Nature tailored enzymes to be highly efficient and selective;^29^ computational design principles are established to develop catalysts and enzymes,^30,31^ exploiting structural^32^ and dynamical^33^ information to optimize reactivity.

There are three principal isoforms of Ras: KRas, HRas, and NRas.^2^ The differences between these are mainly related to the localization and trafficking of the proteins to reach their signaling partners, while their active sites are identical. Importantly, the most frequent oncogenic mutations correspond to only three active site residues: Gly12, Gly13, and Gln61, totaling to over 97% of all Ras mutations.^3^ Here, we focus on the key oncogenic mutation site, Gly12. G12D is overwhelmingly the most frequent Ras mutation, present in half of the Ras positive cancers.^2^ We also investigated G12C, which provides an option for covalent inhibition^34,35^ with two drugs Sotorasib (AMG510) and Adagrasib (MRTX849) currently approved by the FDA.^36−40^

Experimentally, Ras structures are well-characterized, and transition state (TS) analogues are available in Ras.GAP bound complexes.^41^ We used the Ras.p120GAP complex (PDB ID 1WQ1) as the starting structure for our simulations (Supporting Information, section I).^42^ The active site of Ras (Figure 1B) and the associative phosphate cleavage reaction are also well established.^43^ An essential Mg^2+^ ion coordinates the β- and γ-phosphates,^44^ Ser17, Thr35 of the RAS effector lobe, and two water molecules.^45^ The nucleophilic water molecule is positioned near the γ-phosphate via H-bonding to Gln61:Oε and the Gly60 backbone. Lys16 and the important arginine finger Arg789 of the GAP coordinate the GTP.

**Figure 2:**
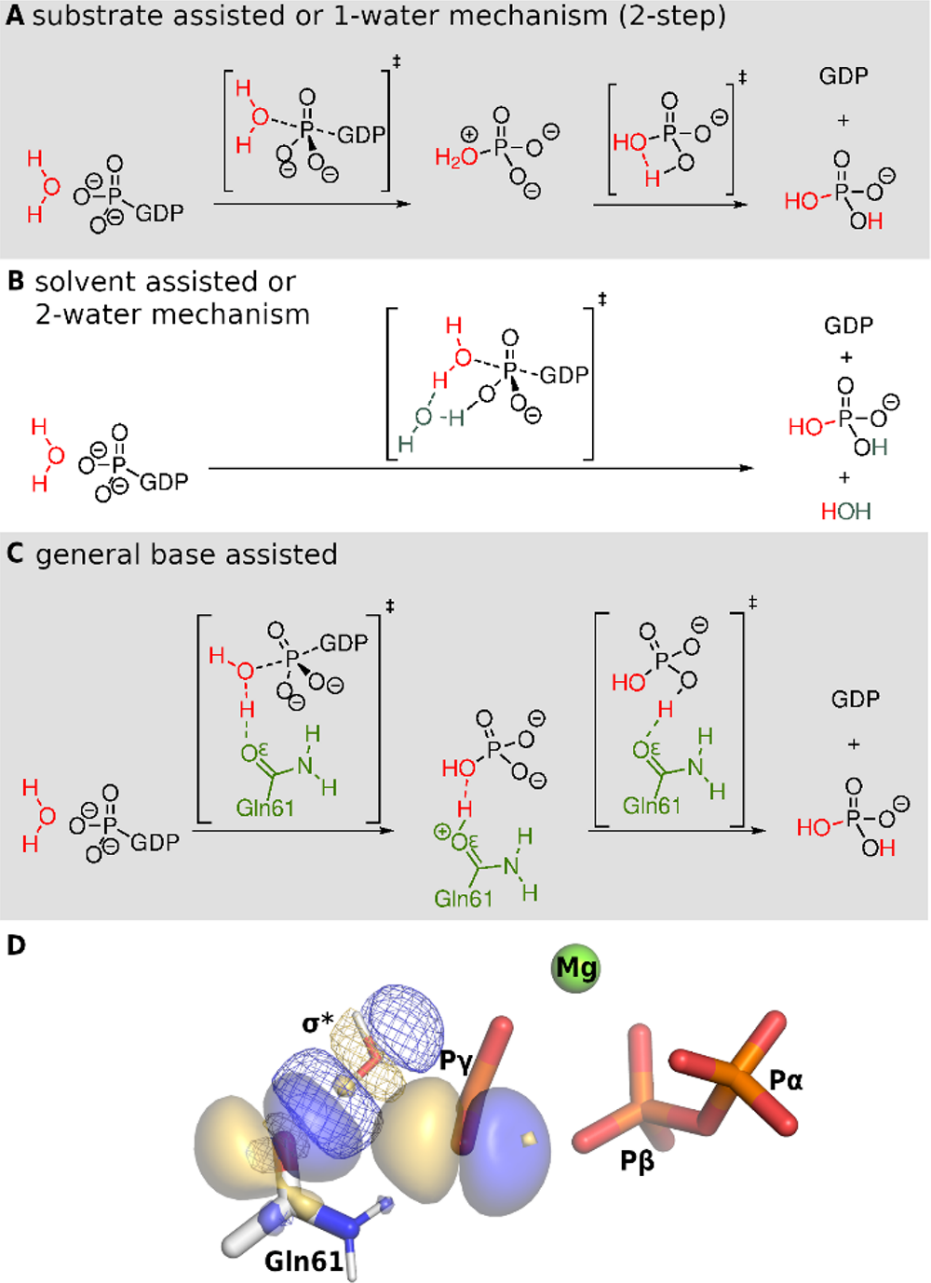
(A–C) Proton transfer alternatives during GTP hydrolysis. (D) Natural bonding orbitals during the phosphate cleavage. Solid surfaces represent occupied NBOs (lone pairs), meshes depict the virtual antibonding orbital of the Wat_nuc_ O–H bond. The electron donation from the axial direction by Oε of Gln61 is more favorable than the donation from the phosphate oxygen.

The catalytic mechanism, however, leaves many questions unanswered. The main controversy involves the proton transfer mechanism of the GTP hydrolysis reaction.^46,47^ Upon hydrolysis, the nucleophilic water gets deprotonated, while one of the oxygens of the formed inorganic (dihydrogen)phosphate (P_i_) gets protonated. Potential mechanisms were proposed to be (i) a direct transfer (substrate assisted or 1 water, 1W mechanism, Figure 2A), (ii) via an additional water molecule (solvent assisted or 2 water, 2W mechanism, Figure 2B), or (iii) catalyzed by a basic protein residue (general base assisted, Figure 2C).^46^

**Figure 3:**
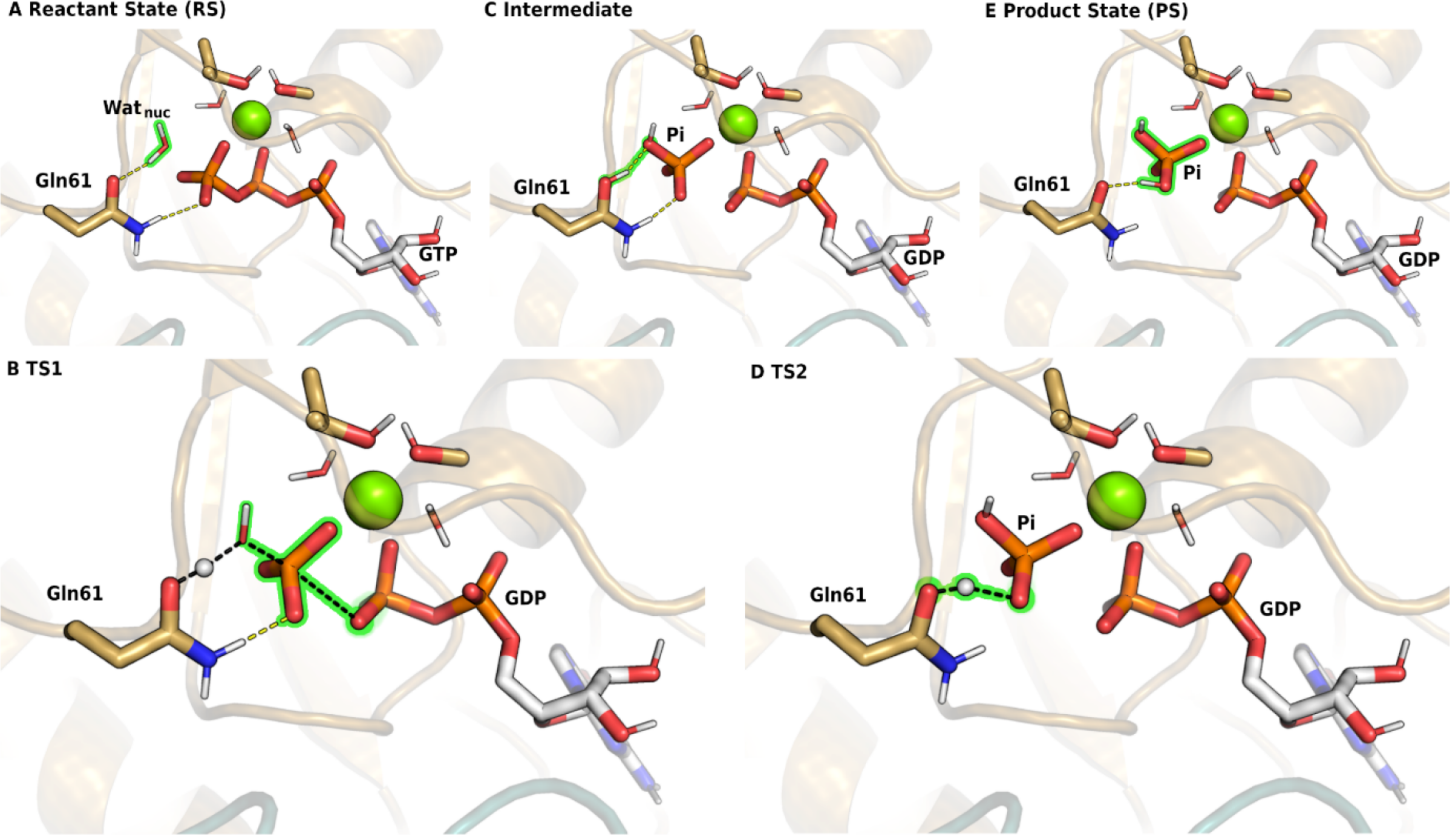
Stationary points along the wild-type Ras.GAP GTP hydrolysis. Breaking and forming bonds (black dashes) and hydrogen bonds (yellow dashes) are depicted. (A) Reactant state. (B) First transitions state. (C) Intermediate with protonated Gln61. (D) Second transition state. (E) Product state of a bound GDP + P_i_. For clarity, nonpolar hydrogens are omitted.

Despite multiple studies proposing reaction mechanisms for wild-type (WT) Ras, very little is known about how detrimental changes in enzyme activity are induced by oncogenic mutations. Experimental evidence, including kinetic rate measurements, are nevertheless available for WT and mutant Ras proteins,^12,24,48^ pointing to the loss of catalytic activity due to the impaired rate of hydrolysis. Computational studies elaborated on the changes in the reactant state (RS, Figure 3A) Ras.GTP complex structures upon Gly12, and Gln61 mutations,^49−55^ including in-depth analysis of the changes in atomic charges and the polarization of the active site before the reaction.^56^ However, calculations to evaluate the influence of the important oncogenic changes on the reaction mechanism are missing.

## Results and Discussion

To assess the structural changes caused by the key oncogenic mutations of Gly12, G12C, and G12D, we analyzed classical molecular dynamics (MD) trajectories (Supporting Information, section II). In general, the Cys12 substitution causes less disruption in the active site conformations, while the Asp12 substitution induces more notable changes, such as weakening the interaction of the GTP with the Switch I loop (Table S2–3). Importantly, both mutations affect the contact with Gln61, and the interactions with the side chain are about 50% present during the simulations, while with Gly12, such interactions are absent. Given the essential role of Gln61 in the hydrolysis, this interaction is likely to contribute to the diminishing activity. The stabilizing role of Gln61 in the H-bonding pattern in the RS was previously also highlighted.^57^ Accordingly, G12C and G12D mutations were found to induce conformational changes in Gln61.^49^ Rearrangements of water molecules were observed at the active site, consistently with our MD simulations. The disturbance of the water distribution was also observed in many Gln61 mutants.^58^ Nevertheless, no major structural changes were otherwise identified in the active site. Therefore, these changes alone may not account for the major loss of activity in the Gly12 mutants.

To reveal how these key oncogenic mutations act on the catalytic pathway, we first explored the WT Ras.GAP reaction mechanism, including the proton transfer steps using QM/MM free energy calculations (Supporting Information, section IV). We found that the substrate assisted transfer (1W) to the phosphate (Figure 2A) has a large barrier (Figure S1) and it is likely unfeasible due to the orbital orientation of the breaking bond. Figure 2D depicts two lone pair Natural Bonding Orbitals (NBOs) that may donate electron density toward the unoccupied O–H anti-bonding orbital of the nucleophilic water (Wat_nuc_) to demonstrate the significant advantage of the orientation provided by Gln61. The perturbation of the Gln61:Oε lone pair is two orders of magnitude higher than that of the lone pair of the O3γ (Table S5). We therefore included additional water molecules (Figure S2) to facilitate this proton transfer (Figure 2B); however, these attempts also produced a high barrier (Figure S3).

The importance of Gln61 was recognized by early studies,^59,60^ by activating the Wat_nuc_. The amide-imide tautomerization of the Gln61 side chain was suggested by Nemukhin et al.^61,62^ and Warshel et al.^63^ The tautomerization was backed with vibrational spectroscopy results, although for a photocatalytic reaction.^64^ We used constrained QM/MM minimizations to explore the mechanism to form the phosphate product by tautomerizing Gln61 into an imide. Our attempts to establish an intermediate with the imide form of Gln61 failed, and the Nε regained the proton from the phosphate.^65^ Instead, we obtained the lowest barrier energy minimized path via a transient proton transfer to the key Gln61 residue via Gln61:Oε (Figure 2C). In our simulations, the rate-determining step is the protonation of the inorganic phosphate by the transient GlnH^+^. The tautomerization and the base catalysis was also compared using QM/MM umbrella sampling simulations for the related GTPase, Arl3, whereby the GlnH^+^ intermediate was found to be more stable than the imide.^66^ A similar mechanism was proposed recently by Nemukhin et al. for the catalytic mechanism of Ran GTPase^65^ and was also listed as one of the possible options for the Rho GTPase mechanism by Blackburn et al.^67^ Previous calculations based on the PM3 semiempirical method suggested that the Gln61 is not basic enough,^68^ which underlines the need for high-level QM methodology. In an NMR study of differently protonated intermediates, it was suggested that even the GDP can be transiently protonated.^69^ Gln61 was suggested to serve as a base in very early studies,^70^ although we find that the proton transfer is tightly coupled to the phosphate cleavage and does not take place *a priori* as a separate step.

The five stationary points of our proposed mechanism are depicted in Figure 3. The first transition state (TS1) corresponds to the nucleophilic substitution on the phosphorus and the proton transfer from Wat_nuc_ to the Gln61 (Figure 3B). The obtained intermediate (Figure 3C), characterized by the protonated Gln61, is in strong H-bonding interaction with the newly formed inorganic phosphate. This interaction breaks during the second, rate-limiting transition state (TS2, Figure 3D), whereby the phosphate rotates to enable the proton transfer from the Oε of the Gln61 sidechain. In the direct product complex (PS, Figure 3E), the P_i_ remains in coordination with the Mg^2+^.

**Figure 4:**
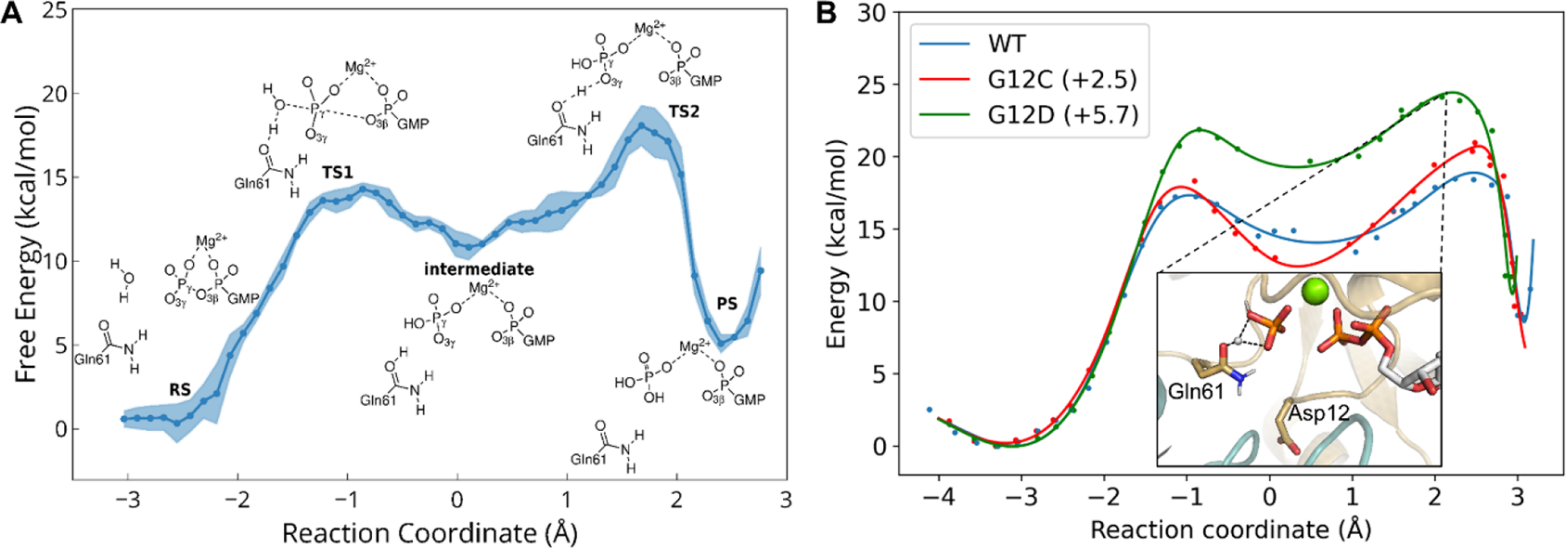
(A) Free energy reaction profile from string calculations projected along reaction coordinate, as defined in the Supporting Information, section VII. Shades depict the estimated variation of the profile along the energy axis. Stationary structures are drawn schematically. (B) QM/MM energy from constrained minimizations of the WT (blue), G12C (red), and G12D (green) Ras using the reaction coordinate, as defined in the Supporting Information, section VII. TS2 for the G12D mutant path is depicted in the inset. Final single point energies are calculated at the ωB97M-V/cc-PVTZ level of theory.

The optimized reaction profile was used as the starting point for the finite-temperature string method (Supporting Information, section VII). The free energy profile is reconstructed using WHAM,^71^ and it is depicted, along with the estimated uncertainty, in Figure 4A. The overall barrier that corresponds to the second, rate-determining step is 18.1 ± 1.6 kcal/mol, in good agreement with experimental rates (Table 1). It is worth to point out, however, that experimental assays measure the enzymatic reaction rate in tandem with other steps, such as complex formation or product release. For this comparison, it is generally assumed that the chemical step is rate determining. Nevertheless, despite that the current mechanism seems highly likely, we cannot exclude larger structural changes that might accompany, or prelude, the second proton transfer. This is also possible, considering available structural data, as GDP-bound Ras has a distinct switch I-II domain conformation,^27^ and such a conformational change must take place after the cleavage of the γ-phosphate. However, the current QM/MM-based methods would not be able to capture such significant structural rearrangements, even if the timescale is fast, and future work will be needed to evaluate this mechanism.

**Table 1:** Computational and Experimental Activation Barriers and Reaction Rates of GTP Hydrolysis Catalyzed by Ras.GAP

	activation barrier (kcal/mol)			reaction rate (s^–1^)		
method/source	WT	G12C	G12D	WT	G12C	G12D
QM/MM free energy calculations^a^	18.1			1.1		
QM/MM minimization and SP^b^	18.5	21.0	24.1	0.6	9.5 × 10^–3^	7.0 × 10^–5^
Wey et al.^72^	16.4	23.1	24.3	1.8 × 10^1^	3.2 × 10^–4^	5.0 × 10^–5^
Hunter et al.^48^	19.0	22.1	21.2	4.3 × 10^–2^	2.0 × 10^–4^	8.9 × 10^–4^
Johnson et al.^73^	21.4		24.4	5.1 × 10^–3^		3.7 × 10^–5^

a Finite temperature string free energy calculations.

b Potential energies obtained from constrained optimizations, followed by higher lever single point calculations. Rates and barriers were interconverted assuming first-order kinetics at 310 K, except for the experiments by Hunter et al, which were done at 293 K.

Subsequently, using our WT mechanism as the starting point, we also investigated the reaction paths for the G12D and G12C replacements. Reaction barriers from constrained QM/MM minimizations along the reaction path, then the final QM/MM energies were recalculated with the ωB97M-V functional (Figure 4B, green and red, respectively). The obtained potential energy barriers are in good agreement with experimental rates (Table 1); however, the accuracy could be further improved by performing free energy calculations.

G12C presents a smaller change of 2.5 kcal/mol in the activation barrier of the Ras.GAP reaction in accordance with the smaller structural changes observed during the MD simulations. It mainly increases the barrier of the second step, required to complete the proton transfer to the inorganic phosphate. G12C was found to slow down both p120GAP-activated^48^ and NF1-activated^28^ hydrolysis rates.^74^ On the other hand, the G12D barrier is higher than the WT for both steps, increasing the barrier by 5.7 kcal/mol. The comparison of the NBO charges reveals the modest changes. In the first reaction step, the electron at the attacking oxygen is slightly reduced by the G12D mutation (+0.008), and it is not observed for G12C (−0.001). This lowers its nucleophilicity and is thereby a possible explanation for the observed barrier increase. At the same time, the NBO charge of the γ-phosphorus decreases by 0.011, making it a worse electrophile. In the case of the G12C mutation, this change is smaller (−0.007) and the barrier does not change significantly compared with the WT (Table S8). While experimental measurements of the hydrolysis rate are challenging, and a coupled enzyme is typically needed to assess the forming P_i_ concentration, our results have a good agreement with the reported changes in the rates (Table 1). Wey et al. used a comprehensive kinetic model parameterized by measurements, and they found that the hydrolysis step is more effected by the G12D replacement than by G12C.^72^

The general base-assisted mechanism is also supported by experimental findings that the Q61E mutant Ras has an increased intrinsic GTPase activity.^68,75^ Furthermore, Gln61 of the switch II loop is conserved (Figure S4) among small GTPases, and these are often linked to disease. Similarly to NRas, in which Q61 mutations are the most frequent in melanoma, Q209P mutants of GNAQ are also associated to melanoma.^76^ The Q64L mutation was found to activate Rheb identified in tuberous sclerosis complex disease.^77,78^ Arl2 and Arl3 are known to have leucine replacements in the corresponding Gln positions 70 and 71, respectively, causing vision impairment.^79,80^ RhoH, a Rho isoform, is expressed in hematopoietic cells, and its altered expression levels were observed in lymphomas and leukemias. RhoH lacks Gln61 and Gly12, and it is known to have no GTPase activity.^81^ Rab25, also known as Rab11c, of the Rab family, is found at high levels in epithelial tissue; its altered expression and mislocalization are associated with aggressively metastatic cancer.^82^ Compared to other Rab11 isoforms, it includes a Gln-to-Leu mutation (Figure S4), rendering Rab25 proteins GTPase deficient.^83^ In Rap1a, the conserved glutamine residue was found to be replaced by an asparagine from its GAP to enable catalytic activity.^84^

In other phosphatases, a stronger Brønsted base is often used. For example, the GTPases hGBP1^85^ and FeoB^86^ as well as the ATP dependent myosin motor domain^87^ use glutamate as a base, accessed through a proton relay. Analogous roles for sidechain-assisted proton transfer also involves aspartate (e.g., for dUTPase^88^) or histidine residues (for RNase H, RNase T, or RuvC)^89^ in other phosphate cleaving enzymes. Nevertheless, the identification of the base is often a challenge for mechanistic studies.

**Figure 5:**
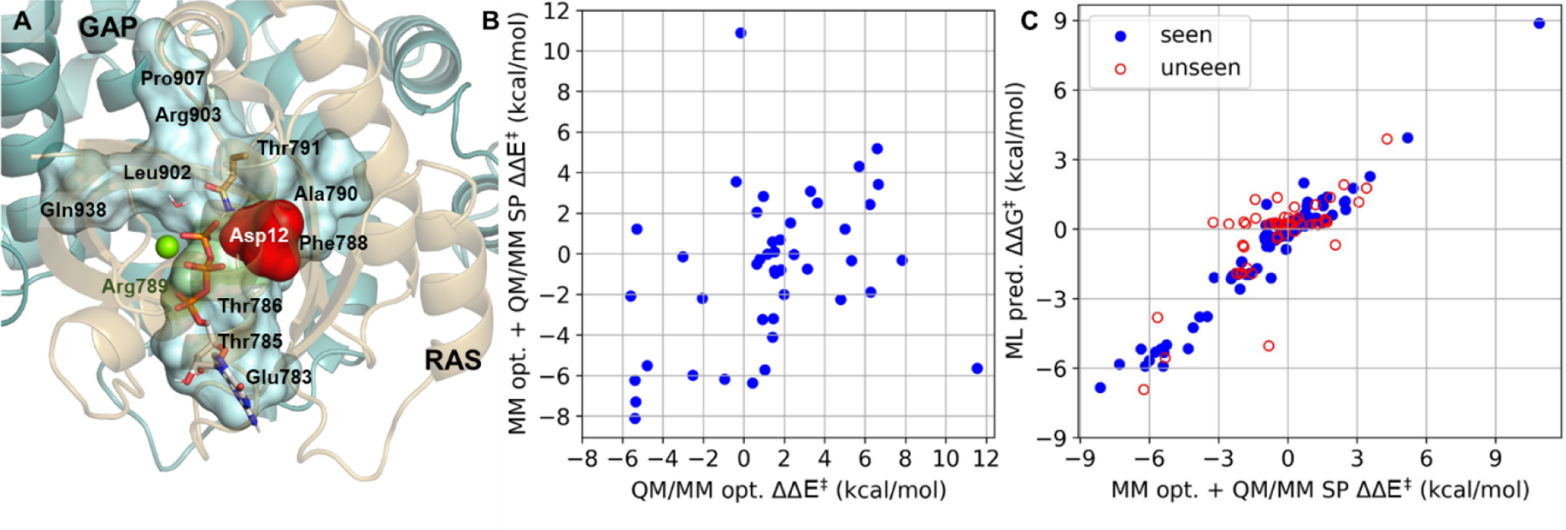
(A) Selected GAP mutation sites (cyan surface, black labels) around the GTP (sticks) pocket in the G12D (red surface) Ras (gold cartoon).p120GAP(blue cartoon) complex. The arginine finger (green surface) is also highlighted. (B) Validation of the barrier estimation protocol against full QM/MM reaction path scans. (C) Gradient boosting regression performance trained on the values from the screening protocol: 70% training data (blue dots) and the 30% validation set (red circles).

With the optimized reaction pathway available to model the loss of Ras activity, we next investigated the possibility to reactivate oncogenic Ras G12D by redesigning selected GAP residues. We focused on the G12D mutant, as it is the most frequent mutation among all Ras isoforms and, unlike G12C, there are no approved targeted therapies. We identified 10 mutation sites for GAP that are closest to the active site and mutated Ras Asp12 residue, not including the Arg finger (Figure 5A, Supporting Information, section X). To reduce the high computational costs for full reaction pathway optimization of the 190 possible single GAP mutants, we developed a simplified screening protocol (Supporting Information, section X) to estimate the barrier height with the modified GAP chains (Table S9). This approach uses the initial pathway from our QM/MM optimized mechanism for G12D Ras. For every point along the path, we optimize the geometry using a simplified QM/MM energy evaluation, where the QM atoms involved in the reaction are held in place, and all MM atoms are allowed to be reoptimized. Finally, the energies of the highest TS and the RS are calculated via QM/MM single point calculations. We evaluated the accuracy of this protocol by calculating the reaction profile for selected 45 GAP mutants in complex with G12D Ras (Figure 5B, Table S10 and Supporting Information, section XI) resulting in a 0.36 correlation and average error (RMSE) of 4.8 kcal/mol for the barrier height. Considering the reasonable correlation and that the barrier heights can change over 15 kcal/mol, these calculations are useful to reduce the number of potential mutants, significantly decreasing the computational costs. The best predicted GAP mutants are subsequently fully QM/MM minimized for better accuracy.

Ultimately, we created a machine learning model using extreme gradient-boosting regressor to further enable large-scale screening (model details in Table S11). Every GAP variant was represented by a sequence of the 10 selected residues, and every residue was described by three simple descriptors, including the charge, dipole moment, and the number of heavy atoms (see Table S12). With *k*-fold cross-validation, the regression model performs excellently on unseen data (correlation: 0.76, RMSE: 1.75 kcal/mol, Figure 5C) enabling ultrafast prediction of the modified reaction barriers.

Our results suggest that the effects of oncogenic G12D mutations can be overcome by appropriate GAP mutations, and over 6 kcal/mol changes in the barrier height are possible (Table 2). The most apparent patterns among the favorable GAP mutants are observed with ionic residues (Table S9). Close to the phosphate end of the active site and the Switch II loop, the removal of the positive charge of Arg903 or the introduction of a negative charge at Leu902 or Pro907 is highly beneficial for decreasing the reaction barrier. Interestingly, if the modeled Ras.RGS3 complex^28^ is aligned to the Ras.p120GAP, the approximate positions of Arg903 and Pro907 are taken up by an Asp and Asn residue, respectively (Figure S6). In the region near Glu783 and Thr785, the opposite trend is observed; more positively charged substitution is favorable to promote GTP hydrolysis.

**Table 2:** Top GAP Mutants Obtained for G12D Ras Activation Using QM/MM Minimizations^a^

Ras	GAP	Δ*E*^‡^_ωB97M-V_	ΔΔ*E*^‡^_ωB97M-V_	ΔΔ*E*^‡^_opt, b3lyp_	ΔΔ*E*^‡^_scr, b3lyp_
WT	WT	18.5	N/A	N/A	N/A
G12D	WT	24.1	0.0	0.0	0.0
G12D	R903E	17.4	–6.7	–5.4	–8.1
G12D	L902D	17.7	–6.4	–5.6	–2.1
G12D	R903D	18.4	–5.7	–5.4	–6.2
G12D	L902E	18.5	–5.6	–5.3	–7.3
G12D	T785D	19.3	–4.8	–5.3	1.2

a Relative barriers (in kcal/mol) are compared with the G12D Ras/WT GAP complex from minimizations (ΔΔ*E*^‡^_opt_) and from the screening protocol (ΔΔ*E*^‡^_scr, b3lyp_).

The best predicted three single mutants using our simplified MM optimization + QM single point scheme are glutamates, at positions Leu902, Arg903, and Pro907 (Table S9). After QM/MM optimization, the Glu and Asp replacements in the 902 and 903 positions remained to be the most beneficial for the reaction (Table 2), including the costly ωB97M-V calculations. While the full optimization changes some of the results from the simplified protocol significantly, the single point correction does not. Overall, we concluded that, although full minimization is required for better accuracy, the QM level is satisfactory for screening. Importantly, while the G12D Ras mutation slows down the hydrolysis rate by several orders of magnitude (Table 1), in our calculations, optimal GAP mutations can exert an opposite effect.

Our protocol can be further extended to double and multiple mutations. Selected double mutations were also explored. While electrostatic effects are well described in our models, sampling of the conformational space is important and scales poorly. We furthermore assume that binding to Ras is not diminished by the mutations. After QM/MM minimizations of the most promising double mutants, the R903E mutants stand out as the main driver of the effect on the barrier (Table S13). We also trained a regression model based on the single GAP mutants, which can predict the effects of the double mutants very accurately (Figure S7).

In conclusion, we present a novel mechanism for Ras.GAP-catalyzed reaction using QM/MM free energy calculations. We considered alternative proton transfer mechanisms coupled to the phosphate cleavage and identified a transient protonation of Gln61 as the most favorable, in accordance with analogous GTPases.^65,66^ Importantly, the obtained mechanism also allows us to compare reaction rates for two key oncogenic mutations: G12C and G12D. The agreement observed with experimental rates validates the detailed proton transfer steps that involve the crucial Gln61 residue as the transient proton acceptor.

Our mechanism provided a starting point for computational screening to reactivate oncogenic Ras, focusing here on G12D, for which no approved treatment is currently available. To this aim, we designed GAP variants using a stepwise QM/MM-based protocol on 10 selected residues. We explored over 200 sequences, including 190 single point mutations and identified top GAP mutants. Importantly, our obtained barrier heights suggest that re-activation of G12D oncogenic Ras by GAP mutants is a viable approach. We suggest that R903E and L902D GAP are the most promising to decrease the activation barrier in the G12D Ras.GAP complex.

This work is a proof of principle in establishing an approach that can be extended toward designing multiple GAP mutations or even to drug molecules that are capable of restoring the lost catalytic activity due to the oncogenic Ras mutations. Our machine learning models furthermore demonstrate excellent prediction accuracy and can offer a high-throughput screening option to molecular design aiming catalytic re-activation. The multiple layers of the outlined screening approach, from free energy calculations to machine learning regression, enable an affordable scale-up for computational screening, while maintaining the accuracy of the final predictions.

Using our protocol, we also open up novel high-throughput methodologies to aid the computational prediction of small molecule ligands that, instead of inhibiting an enzyme reaction, restore the catalytic activity of disease-causing loss-of-function mutant enzymes.

## Supporting Information Available

Model building, computational details, alternative mechanism, NBO analysis, the screening protocol, machine learning regression, and barrier prediction (PDF)
