# Supplementary material for: Mechanism-Based Redesign of GAP to Activate Oncogenic Ras
Source: J Am Chem Soc. 2023 Sep 8;145(37):20302–10. doi: 10.1021/jacs.3c04330 (PMC10515638; doi:10.1021/jacs.3c04330)
Supplement: Supplementary file 1 — ja3c04330_si_001.pdf [file ja3c04330_si_001.pdf]

Supporting Information

Mechanism-based Redesign of GAP to Activate Oncogenic Ras

Dénes Berta [a], Sascha Gehrke [a], Kinga Nyíri [b,c], Beáta G. Vértessy [b,c], Edina Rosta [a]*

[a] Department of Physics and Astronomy, University College London, Gower Street, London WC1E 6BT

[b] Institute of Enzymology, Research Centre for Natural Sciences, Magyar tudósok körútja 2., Budapest, 1117, Hungary

[c] Department of Applied Biotechnology and Food Science, Budapest University of Technology and Economics, Budafoki út 6-8, Budapest, 1111, Hungary

I. Model Building

The PDB structure 1WQ1 was used as a starting structure.[1] The GDP was completed by a third phosphate in the direction of the AlF_3 to form a GTP. G12 mutations were carried out by CHARMM. The protonation state of the residues was checked with PROPKA 3.1,[2] lysines or arginines were all protonated, glutamates and aspartates were all deprotonated. The single outlier was Glu799 with the predicted pK_a of 8.86, due to its proximity to Glu63, however, it was kept deprotonated as the carboxylate is in the vicinity of Lys803 and is easily solvated by water molecules as well.

The histidine residues were individually checked to determine the protonation state based on their contacts. The delta protonated species was used in all cases as it is slightly more favorable thermodynamically and no strong interactions affect this state.

Table S1. Histidine residues and their protonation states, based on contacts observed in the crystal structure 1WQ1.

Residue ID	Chosen protonation	contact
27	HSD	exposed to water
94	HSD	donates to Tyr137
166	HSD	exposed to water
736	HSD	exposed to water
743	HSD	exposed to water
762	HSD	accepts from Thr989
811	HSD	exposed to water
812	HSD	exposed to water
847	HSD	may accept from Ser824
883	HSD	exposed to water
965	HSD	exposed to water
999	HSD	no H-bonds
1005	HSD	may donate to Ile1001
1021	HSD	exposed to water

II. MD Simulations

Classical MD simulation were carried out using CHARMM36 force field[3] as implemented in NAMD 2.12[4]. A cubic simulation box was constructed by adding 30657 TIP3 water molecules,[5] 102 K^+ and 86 Cl^- ions. Periodic boundary conditions were used in all classical simulations and the particle mesh Ewald method was used for long-range electrostatic interactions with a cutoff of 12 Å.[6] SHAKE algorithm was deployed to constrain the covalent bonds involving hydrogen atoms.[7]

The energy of the system was minimized using the steepest descent algorithm for a total number of 10000 steps, followed by 10 ns canonical equilibration with restrained heavy atoms. Simulations on microcanonical ensemble were then run at 303.15 K via standard MD procedure with a time step of 2 fs. Langevin temperature and pressure control was used with a damping coefficient of 1 ps^{-1}. Each of the WT, G12C and G12D systems were run for 200 ns three times to produce independent replicas.

III. Contact Changes

The residue-residue contacts were calculated for the joint trajectories, sampled at every 10 ps. Contact frequencies were defined along the trajectory by the ratio of frames where a given contact is below a 4.5 Å cutoff for the closest heavy atom distance. The difference in these contact frequencies between a mutant and the WT trajectory is analyzed.

Table S2. Changes in the G12D mutant contact frequencies compared with the WT MD simulations of the Ras-GAP complexes.

Residue 1	Residue 2	WT	G12D	G12D – WT
LEU752	ASP748	0.00	0.99	0.99
ASP/GLY12	THR791	0.01	0.88	0.86
ASP57	THR20	0.06	0.88	0.82
ASP748	LEU751	0.20	0.99	0.79
GLN1020	GLU1015	0.00	0.79	0.79
TYR32	GTP167	0.01	0.74	0.74
GLY947	ASN957	0.06	0.78	0.71
LEU902	TYR32	0.24	0.94	0.70
ALA59	ARG68	0.05	0.68	0.64
ARG68	GLU63	0.07	0.67	0.61
LYS88	HIS743	0.02	0.62	0.60
PHE760	LEU797	0.00	0.60	0.60
CYS745	LYS88	0.10	0.69	0.59
ALA1018	HIS1021	0.00	0.59	0.59
TYR64	ASN911	0.27	0.82	0.55
THR792	ARG789	0.00	0.54	0.54
ILE873	ASN774	0.34	0.87	0.53
ASN888	ARG892	0.01	0.53	0.52
**ASP/GLY12**	**GLN61**	0.45	0.96	**0.51**
VAL744	LYS88	0.00	0.51	0.51
GLN808	PRO867	0.00	0.51	0.51
GLN878	VAL895	0.69	0.18	-0.51
ARG894	TYR32	0.88	0.34	-0.54
THR786	LYS117	0.55	0.01	-0.54
ASP33	ARG789	0.56	0.01	-0.55
THR786	GLY13	0.55	0.00	-0.55
LEU769	LEU797	0.85	0.30	-0.55
ASP33	GTP167	0.78	0.21	-0.57
GLU63	GLU799	0.72	0.13	-0.60
ASN942	TYR32	0.82	0.15	-0.68
ARG1016	SER1013	0.99	0.18	-0.81
ARG1016	LEU1012	1.00	0.18	-0.82
PRO34	ARG789	0.82	0.00	-0.82
GLY1017	LEU1012	0.96	0.14	-0.82
PRO34	GTP167	0.87	0.00	-0.87

Table S3. Changes in the G12C mutant contact frequencies compared with the WT MD simulations of the Ras-GAP complexes.

Residue 1	Residue 2	WT	G12C	G12C – WT
**CYS/GLY12**	**GLN61**	0.45	0.99	**0.54**
PRO34	GTP167	0.87	0.36	-0.51
GLN1019	LEU1023	1.00	0.47	-0.53
ARG1016	GLN1020	0.92	0.39	-0.53
GLN1019	VAL1022	1.00	0.46	-0.54
ARG1016	SER1013	0.99	0.26	-0.73
ARG1016	LEU1012	1.00	0.25	-0.75
GLY1017	LEU1012	0.96	0.18	-0.78

IV. QM/MM Details

Starting snapshots for QM/MM calculations were chosen from the trajectories with the catalytic water in place (Figure 2A and Figure S2). QM/MM minimizations were carried out in a non-periodic fashion, only keeping residues within 25 Å of the Mg^{2+} ion of the pocket, and fixing the position of those farther than 20 Å.

QM/MM reaction scans were run for several reaction paths. The MM region was described by CHARMM36 force field,[3] while for the QM region, the hybrid functional B3LYP[8] was used with the Pople basis set of 6-31+G*.[9,10] Final single point calculations were done with the range separated hybrid functional ωB97M-V[11] at the triple ζ basis cc-PVTZ.[12] Electrostatic embedding was used as well as hydrogen link atoms at the QM/MM interface, as implemented in CHARMM[13] and Q-Chem 4.3.[14,15] The definition of QM/MM interface is specified in Table S4 for the WT Ras-GAP complex.

To determine the reaction free energies and the corresponding minimum free energy pathway of the mechanism, the finite-temperature string calculations [16,17] were performed at the same QM/MM level of theory. Each string iteration consisted of 100 steps/window of QM/MM Langevin dynamics with 1 fs timestep using CHARMM as the MD engine. Each coordinate was constrained using a force constant of 150 kcal mol^{-1} $Å^{-2}$. The free energy profiles and surfaces of the reaction were obtained using the weighted histogram analysis method.[17,18] The first 10% of each simulation window was omitted to let the system adapt to the new bias position. The set of strings was divided into four equal sections, and the projected free energies were calculated by WHAM to estimate uncertainties.

Table S4. Definition of the QM region for the WT Ras-GAP QM/MM calculations. Atoms bordering the interface at both sides are specified by residue and atom name. Wat9130 originates from the solvation box and is only included in calculations probing the solvent-assisted mechanism.

QM side	MM side	comment
GTP C5’	GTP C4’	GTP phosphates
Gly12 Cα	Gly12 N	P-loop glycin
Gly13 Cα	Gly13 C	
Lys16 Cϵ	Lys16 Cδ	counterionic lysine
Ser17 Cβ	Ser17 Cα	Mg^{2+} coordination
Pro34 Cα	Pro34 C	Switch I backbone
Ile36 N	Ile36 Cα	
Gln61 Cγ	Gln61 Cβ	Gln61 sidechain
Ala59 Cα	Ala59 C	Switch II backbone
Gly60 N	Gly60 Cα	
Arg789 Cδ	Arg789 Cγ	arginine finger (GAP)
**Whole residues in the QM region**		
Thr35		Mg^{2+} coordination
Wat1 and Wat8		Mg^{2+} coordination
Wat11		nucleophilic water
Mg^{2+}		catalytic ion
Wat9130		proton transfer partner

V. Orbital Orientation

Natural Bonding Orbitals (NBOs)[19] were calculated as implemented in Gaussian 09 Revision E[20] to represent the orbital orientations prior to the proton transfer from the nucleophilic water (Figure 3B).

Table S5. Summary of NBO analysis related to the proton transfer from the nucleophilic water to suitable acceptors. The perturbation energy of occupied orbitals is shown to the virtual O-H antibonding NBO 1050 in kcal/mol.

NBO	orbital	occupancy	Energy (a.u.)	perturbation energy
10	Pγ -O3γ bonding	1.982	-0.913	0.20
188	O3γ lone pair	1.863	-0.281	0.00
189	O3γ lone pair	1.814	-0.280	0.19
207	Gln61:Oε1 lone pair	1.954	-0.656	10.36
208	Gln61:Oε1 lone pair	1.853	-0.330	30.45
1050	Wat_{nuc}:OH antibonding	0.076	0.472	-

The preference for the proton transfer to the Gln61 is also demonstrated by comparing the reaction paths of the direct and assisted protonation. The substrate assisted pathway has a barrier approx. 30 kcal/mol higher than the one which involves the Gln61 (Figure S1).

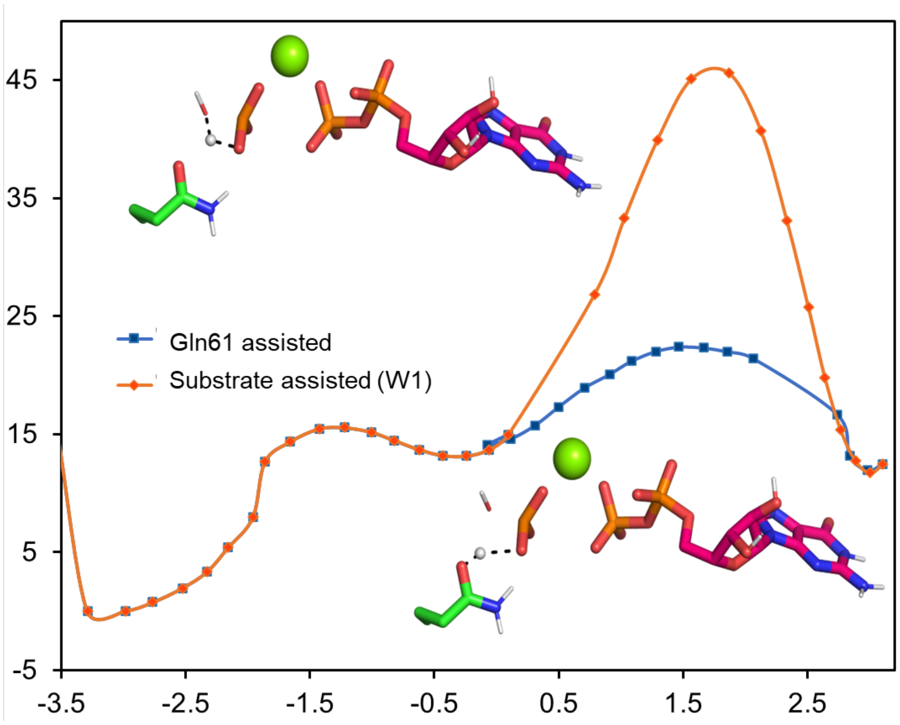

Figure S1. QM/MM minimized paths of the proton transfer to the inorganic phosphate with and without involving Gln61.

VI. Solvent Assisted Mechanism

During the MD simulations, the active site becomes accessible to the bulk water, hence we selected a snapshot (Figure S2) optimal for probing a solvent-assisted mechanism (Figure 2B).

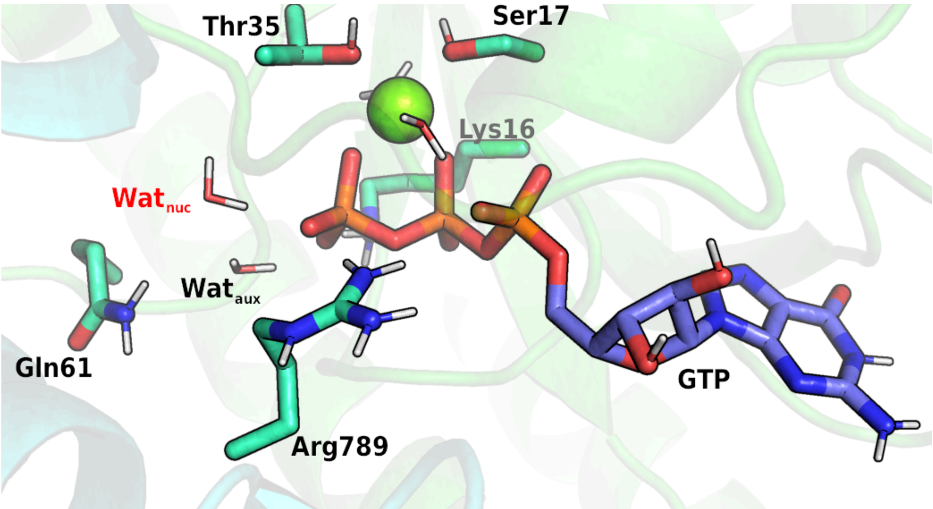

Figure S2. Starting structure for reaction pathways using the solvent-assisted mechanism.

The initial reaction scan was produced using a reaction coordinate that includes the phosphate cleavage, a proton transfer from the nucleophilic water to the auxiliary one, and another from the auxiliary water to the forming phosphate. All steps were concerted. The path was further optimized by the finite temperature string method. The reaction coordinates used are listed in Table S6.

Table S6. Distances included in the finite temperature string calculations of the solvent assisted path.

Atom 1	Atom 2
Wat$_{aux}$:OH2	Wat$_{aux}$:H2
Wat$_{aux}$:OH2	Wat$_{nuc}$:H1
Wat$_{nuc}$:OH2	Wat$_{nuc}$:H1
GTP:O3β	GTP:Pγ
GTP:Pγ	Wat$_{nuc}$:OH2
GTP:O3γ	Wat$_{aux}$:H2
Wat$_{aux}$:OH2	Wat$_{aux}$:H1
Wat$_{nuc}$:OH2	Wat$_{nuc}$:H2
Wat$_{aux}$:OH2	Wat$_{aux}$:H2
Thr35:O	Wat$_{aux}$:H1
Gly60:HN	Wat$_{nuc}$:OH2
Lys16:Hζ3	GTP:O1γ
GTP:O1β	Lys16:Hζ1
Arg789:Hη12	GTP:O3γ
Arg789:Hη11	GTP:O2α
Mg2+	GTP:O2γ
Mg2+	GTP:O2β
Gln61:Hϵ22	Wat$_{aux}$:OH2

The reaction was modelled by 26 windows and 50 string iterations were performed. The average change in the constraint positions in the second half of the string iterations is no greater than 0.01 Å, indicating good convergence. The data obtained in the string iterations was unbiased to obtain the free energy profile depicted in Figure S3. The reaction proceeds via a single step, combining the proton transfers and the phosphate cleavage alike, with a barrier over 30 kcal/mol, significantly higher than that of the general base assisted pathway presented in Figure 4. The reaction coordinate was defined as

$$\chi = d(P\gamma - O3\beta) - d(P\gamma - Wat_{nuc}\colon O) + 0.5d(Wat_{nuc}\colon O - Wat_{nuc}\colon H) - 0.5d(Wat_{nuc}\colon H - Wat_{aux}\colon O) + 0.5d(Wat_{aux}\colon O - Wat_{aux}\colon H) - 0.5d(Wat_{aux}\colon H - O3\gamma)$$

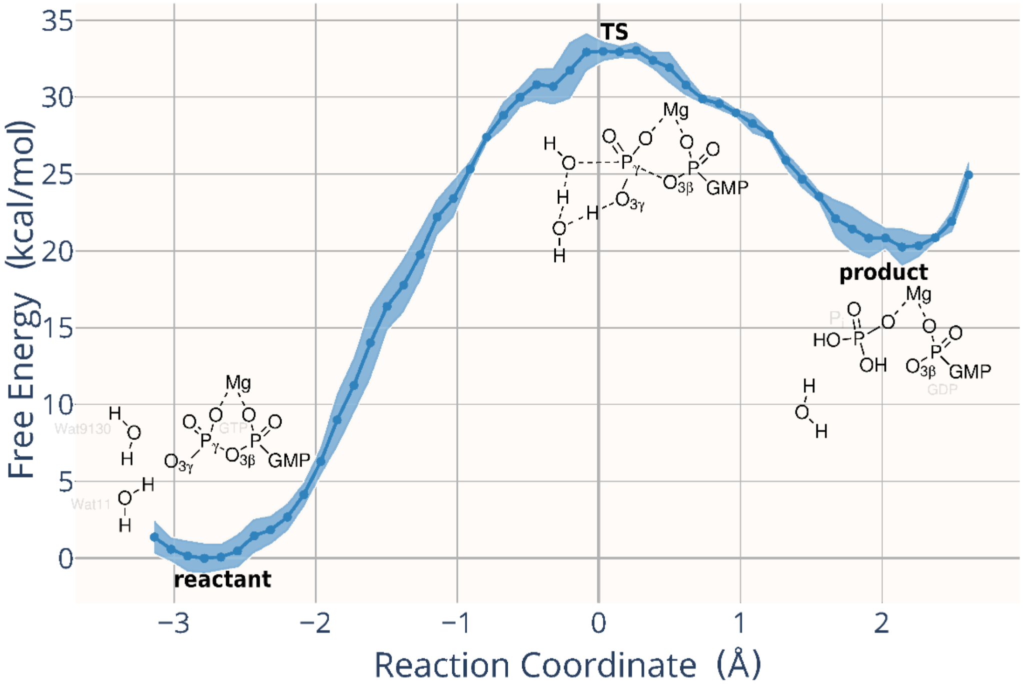

Figure S3. Free energy reaction profile from string calculations projected along reaction coordinate χ. Shades depict the estimated variation of the profile along the energy axis. Stationary structures are drawn schematically.

VII. General Base Assisted Mechanism:

The free energy calculations using the finite temperature string method were constructed with 24 windows, based on the pathway explored by subsequently scanning the two reaction coordinates defined as

$\xi = d(P\gamma - O3\beta) - d(P\gamma - Wat_{nuc}: O) + 0.5d(Wat_{nuc}: O - Wat_{nuc}: H) - 0.5d(Wat_{nuc}: H - O3\gamma).$

The distances involved in the string definition are listed in Table S7.

Table S7. Distances included in the finite temperature string calculations of the general base assisted path.

Atom 1	Atom 2
GTP:O3β	GTP:Pγ
GTP:Pγ	Wat_{nuc}:OH2
Wat_{nuc}:OH2	Wat_{nuc}:H1
Wat_{nuc}:OH2	Wat_{nuc}:H2
Gln61:Oϵ1	Wat_{nuc}:H1
Wat_{nuc}:H1	GTP:O3γ
Gln61:Hϵ21	GTP:O3γ
Thr35:O	Wat_{nuc}:H2
Arg789:Hη12	GTP:O3β
Arg789:Hη12	GTP:O3γ
Arg789:Hη11	GTP:O2α
Lys16:Hζ1	GTP:O1β
Lys16:Hζ3	GTP:O1γ
Gly60:HN	GTP:O1γ
Thr35:HN	GTP:O2γ

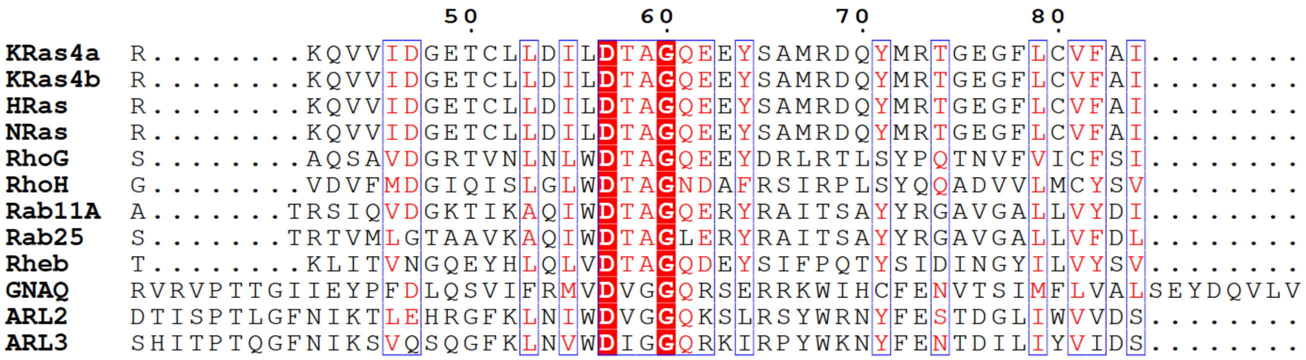

Figure S4. Multiple sequence alignment of the switch II region of GTPases. The catalytic glutamine (Gln61 in Ras isoforms) is conserved, except in the RhoH and Rab25 variants.

VIII. Key distances along the reaction path of the WT, G12C and G12D mutants.

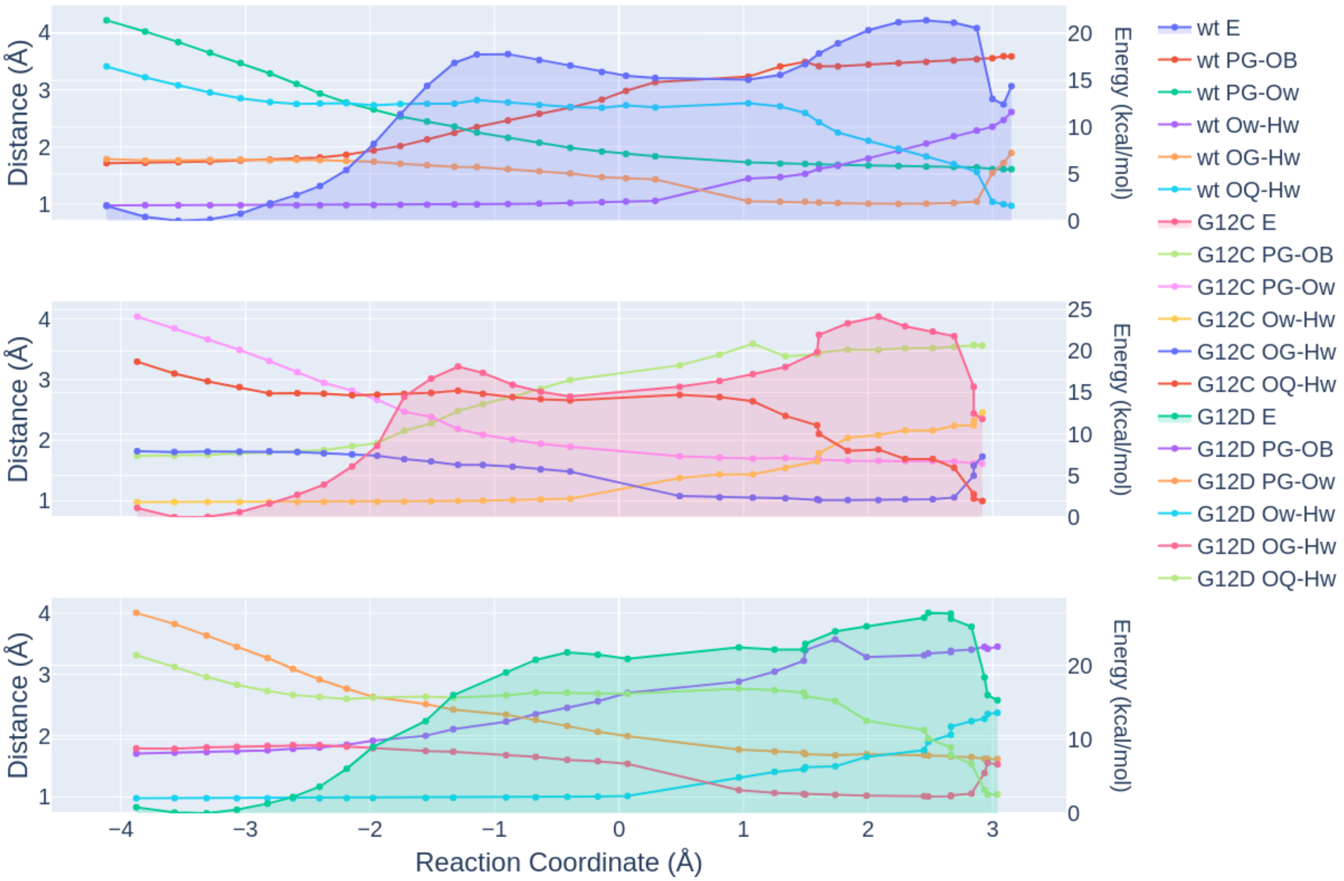

Figure S5. Key distances along the reaction path for the WT, G12C and G12D profiles. The reaction coordinate ξ is displayed in the x axes. QM/MM energies are depicted with shaded curves and measured on the secondary axes. Note that ξ was biased in the corresponding minimizations, not fixing the individual distances.

IX. NBO Charges at Stationary Points

Table S8. Change in the NBO charges of selected atoms calculated at ωB97M-V/6-311G(d,p) level of theory. The values give the changes of the charge in the mutant compared to the WT.

	**reactant**		**TS1**		**TS2**	
	G12C	G12D	G12C	G12D	G12C	G12D
**Gln61:Cδ**	0.012	0.005	0.013	0.007	0.009	0.007
**Gln61:Oϵ1**	-0.006	-0.004	-0.008	-0.008	-0.002	0.000
**Gln61:Nϵ2**	0.002	0.013	0.000	0.013	-0.010	0.005
**Gln61:Hϵ21**	-0.015	-0.017	-0.010	-0.011	-0.002	-0.001
**Gln61:Hϵ22**	0.005	0.005	0.007	0.005	0.012	0.001
**GTP:O3β**	-0.006	0.007	0.000	0.005	0.007	0.004
**GTP:Pγ**	-0.005	0.000	**-0.007**	**-0.011**	-0.010	-0.004
**GTP:O1γ**	0.001	0.003	0.000	0.001	0.001	0.003
**GTP:O2γ**	-0.003	-0.007	0.003	0.004	0.001	-0.001
**GTP:O3γ**	0.006	-0.001	0.006	0.004	-0.010	-0.008
**Wat11:OH2**	0.004	0.003	**-0.001**	**0.008**	0.003	0.000
**Wat11:H1**	-0.001	0.005	-0.001	0.008	-0.003	0.005
**Wat11:H2**	0.001	-0.001	-0.005	-0.007	-0.004	-0.001

X. Barrier Calculation Protocol

The development of our virtual screening procedure was based on the QM/MM minimized reaction path for wildtype (WT) GAP and G12D Ras, the structure and wavefunction of the QM region was taken for the G12D-Ras wt-GAP reaction path. This enabled a quick optimization of the MM region, keeping the QM wavefunction unaltered, along the original reaction path – represented by 33 windows – until convergence was reached.

The defined center of the reaction site via the γ-phosphate, and the sidechains of Gln61 and Asp12. We then identified 11 residues that have sidechain atoms within 10 Å of this center. Our final selection did not include the arginine finger, because of its catalytic importance.

The GAP point mutations were carried out by CHARMM42b2. To account for potential other conformations, three rotamers were generated for all rotatable bonds of the new sidechain additionally, clashes (<1 Å) were eliminated and rotamers over 10 kcal/mol compared to most stable conformer were discarded. The remaining structures were subjected to QM/MM single point calculations. The lowest barrier rotamer was finally selected to represent the single point mutation. Double mutations used the combination of the single mutation conformers.

Table S9. Calculated change in the barrier height for single GAP mutants relative to the G12D Ras.p120GAP system. Values are obtained using the MM-QM/MM protocol and are in kcal/mol.

	mutation site									
	**Glu783**	**Thr785**	**Thr786**	**Phe788**	**Ala790**	**Thr791**	**Leu902**	**Arg903**	**Pro907**	**Gln938**
**Ala**	-2.04	0.64	0.23	0.14	0.00	-0.14	0.12	-4.11	-0.32	0.03
**Arg**	-3.20	-3.23	-0.53	0.84	-0.15	1.53	4.30	0.00	2.43	2.48
**Asn**	-1.67	0.06	-0.28	0.29	-0.14	0.85	0.25	-6.18	0.00	-0.01
**Asp**	0.73	1.22	2.51	-1.82	1.70	0.82	-2.08	-6.23	-5.25	-0.95
**Cys**	-2.20	-0.19	-0.11	0.52	-0.23	0.83	-0.51	-5.73	-0.54	0.37
**Gln**	-2.41	-0.46	-1.01	0.57	0.00	1.69	-0.83	-5.41	-0.08	0.00
**Glu**	0.00	1.49	-0.52	-0.66	0.60	1.21	-7.30	-8.12	-6.36	-2.55
**Gly**	-2.52	-0.16	0.44	-0.26	-0.95	-0.13	-0.20	-5.01	-0.70	0.09
**Hsd**	-1.80	-0.06	-0.99	0.54	0.36	0.29	0.41	-0.82	-1.95	0.72
**Ile**	-2.20	-1.39	1.08	0.00	-0.51	2.43	0.09	-5.37	0.02	1.24
**Leu**	-1.78	0.48	-0.02	0.28	-0.08	3.55	0.00	-5.31	-0.26	1.17
**Lys**	-2.46	-0.55	-0.45	1.02	0.04	1.59	5.19	2.06	3.08	1.70
**Met**	-2.28	0.44	0.68	0.68	-0.84	1.84	0.39	-5.99	-0.44	1.67
**Phe**	-1.54	1.92	-0.18	0.00	-0.31	-0.92	-0.96	-5.64	-1.90	-0.45
**Pro**	-2.21	-1.90	-0.29	0.39	1.31	1.60	0.28	-0.73	0.00	0.19
**Ser**	-1.97	0.34	-0.50	0.43	-0.34	0.10	-0.69	-5.38	-0.93	0.17
**Thr**	-1.65	0.00	0.00	-0.12	-0.19	0.00	0.51	-4.32	-0.80	0.14
**Trp**	-1.77	-0.78	0.25	-0.75	0.53	3.41	0.69	-3.82	-1.99	1.49
**Tyr**	-1.34	0.39	0.30	0.12	1.02	2.83	10.89	-3.49	-2.01	-1.41
**Val**	-2.25	0.64	0.10	-0.45	-0.43	0.72	-0.04	-5.51	-0.14	0.46

XI. Validation of the screening protocol

45 of the 190 screened GAP mutants were fully minimized using QM/MM. The mutants were chosen to include the 9 lowest and the 9 highest predicted barriers, as well as randomly selected other examples. While our top predicted mutants generally performed well except for one of them (R903F), additional top performing mutants can be further evaluated as needed.

Table S10. Effect on the hydrolysis barrier of single mutant GAP proteins as predicted by our simplified screening protocol vs QM/MM optimization of windows. Values are in kcal/mol. The RMSE between the two series is 4.82 kcal/mol.

Mutant	Screening	QM/MM minimization
A790E	0.60	1.42
A790F	-0.31	7.84
A790I	-0.51	0.64
A790R	-0.15	-3.02
A790S	-0.34	5.32
E783I	-2.20	-2.05
E783R	-3.20	1.45
E783V	-2.25	4.80
F788W	-0.75	3.14
L902D	-2.08	-5.60
L902E	-7.30	-5.35
L902F	-0.96	1.56
L902I	0.09	1.52
L902K	5.19	6.60
L902N	-0.83	1.79
L902Q	-0.83	1.51
L902R	4.30	5.70
L902V	-0.04	2.49
L902W	0.69	1.79
L902Y	10.89	-0.15
P907E	-6.36	0.44
P907K	3.08	3.30
P907L	-0.26	0.80
P907R	2.43	6.23
P907T	-0.80	1.84
P907Y	-2.01	1.99
R903A	-4.11	1.42
R903C	-5.73	1.04
R903D	-6.23	-5.38
R903E	-8.12	-5.39
R903F	-5.64	11.54
R903K	2.06	0.64
R903M	-5.99	-2.53
R903N	-6.18	-0.96
R903V	-5.51	-4.78
T785D	1.22	-5.30
T785P	-1.90	6.27
T785R	-3.23	0.92
T786D	2.51	3.63
T786L	-0.02	1.17
T791E	1.21	5.00
T791L	3.55	-0.39
T791R	1.53	2.32
T791W	3.41	6.66
T791Y	2.83	0.96

XII. Machine Learning Regression Details

The regression model was initialized with the following parameters:

Table S11. XGboost regressor parameters.

Parameter	value
**Number of estimator trees**	1000
**Learning rate**	0.01
**Max depth**	4
**L1 regularization term**	0.5
**Subsample ratio**	0.7
**Column subsample ratio**	0.8
**Metric**	MSE

Table S12. Descriptors used for amino acid residues: *q*: formal charge; *μ*: dipole moment (in Debye); *n*: number of heavy atoms in the sidechain.

Residue	Q	M	N
**Ala**	0	0.105197	1
**Arg**	1	1.441688	7
**Asn**	0	0.959357	4
**Asp**	-1	0.861119	4
**Cys**	0	0.354682	2
**Gln**	0	0.84597	5
**Glu**	-1	1.213579	5
**Gly**	0	0.667133	4
**Hsd**	0	0.980225	6
**Ile**	0	0.10726	4
**Leu**	0	0.115873	4
**Lys**	1	2.312181	5
**Met**	0	0.205317	4
**Phe**	0	0.122808	7
**Pro**	0	0.249627	3
**Ser**	0	0.560862	2
**Thr**	0	0.568612	3
**Trp**	0	0.36237	10
**Tyr**	0	0.453853	8
**Val**	0	0.109895	3

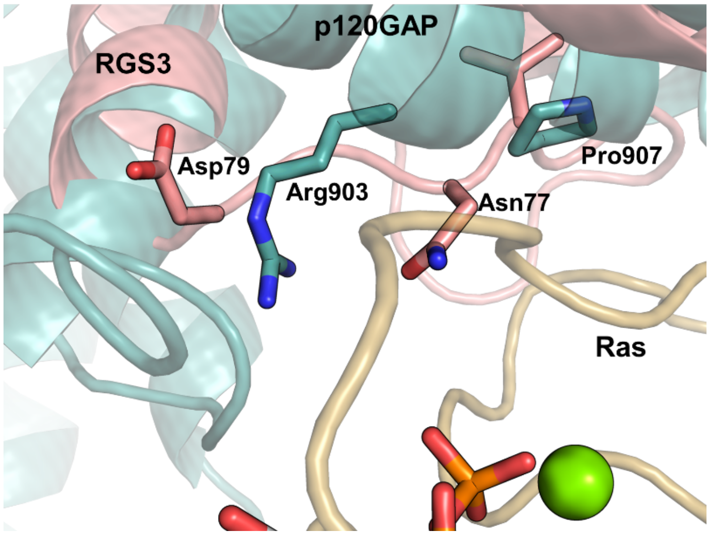

Figure S6. Alignment of the Ras.p120GAP complex (based on PDB 1WQ1) with the RGS3 domain (2OJ4). Important catalytic residues Gln61 and Arg789 are displayed in sticks. GAP mutation site Arg903 are partially covered in the RGS3 structure by Asn77 (sticks).

XIII. Double Mutants

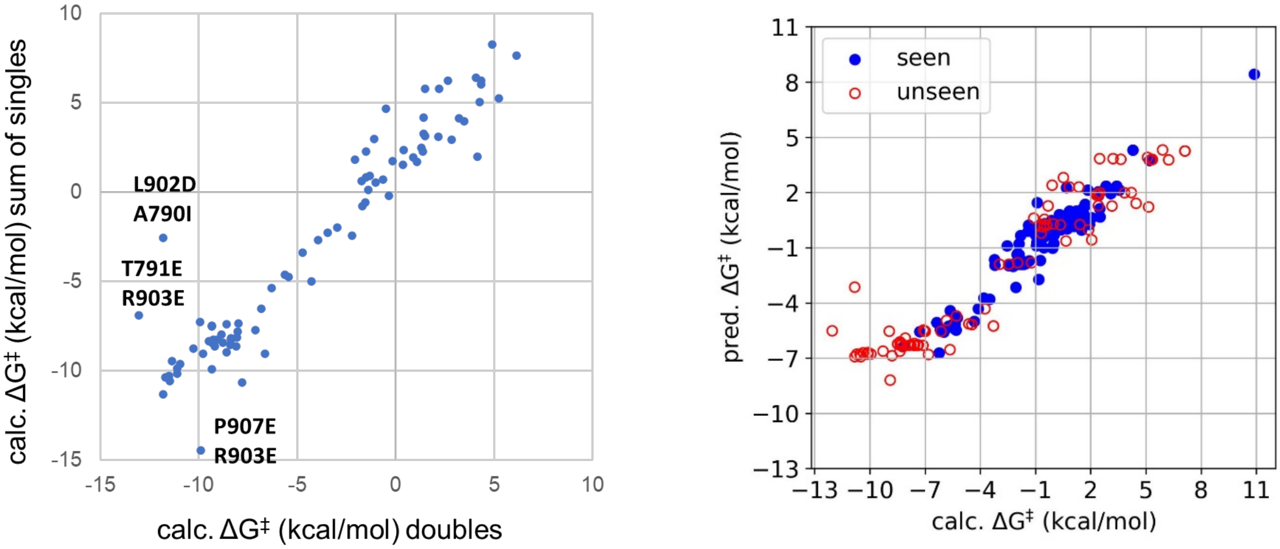

Figure S7. Left: comparison of double mutant prediction with the sum of the two corresponding single mutation's effects. Right: performance of the XGboost regression trained at single mutants (blue dots) predicting a set of 85 double mutants.

Table S13. Effect on the hydrolysis barrier of double mutant GAP proteins as predicted by our simplified screening protocol vs QM/MM optimization of windows. Values are in kcal/mol. The RMSE between the two series is 5.26 kcal/mol.

Double mutants		Screening	QM/MM minimized
**E783R**	R903E	-11.81	-13.46
T791E	R903E	-13.04	-11.80
E783V	R903E	-11.71	-10.95
E783I	R903E	-11.53	-9.28
L902D	A790I	-11.82	-1.25
A790Y	L902K	2.64	-0.60
T785D	A790Y	-1.51	0.88
T785D	A790E	-2.09	5.53
T785D	L902K	4.09	8.05
A790E	L902K	1.49	9.58

References

(1) Scheffzek, K.; Ahmadian, M. R.; Kabsch, W.; Wiesmüller, L.; Lautwein, A.; Schmitz, F.; Wittinghofer, A. The Ras-RasGAP Complex: Structural Basis for GTPase Activation and Its Loss in Oncogenic Ras Mutants. *Science (1979)* **1997**, *277* (5324), 333–339. https://doi.org/10.1126/science.277.5324.333.

(2) Olsson, M. H. M.; Søndergaard, C. R.; Rostkowski, M.; Jensen, J. H. PROPKA3: Consistent Treatment of Internal and Surface Residues in Empirical p K a Predictions. *J Chem Theory Comput* **2011**, *7* (2), 525–537. https://doi.org/10.1021/ct100578z.

(3) D. MacKerell, A.; Bashford, D.; Bellott, M.; L. Dunbrack, R.; D. Evanseck, J.; J. Field, M.; Fischer, S.; Gao, J.; Guo, H.; Ha, S.; Joseph-McCarthy, D.; Kuchnir, L.; Kuczera, K.; T. K. Lau, F.; Mattos, C.; Michnick, S.; Ngo, T.; T. Nguyen, D.; Prodhom, B.; E. Reiher, W.; Roux, B.; Schlenkrich, M.; C. Smith, J.; Stote, R.; Straub, J.; Watanabe, M.; Wiórkiewicz-Kuczera, J.; Yin, D.; Karplus, M. All-Atom Empirical Potential for Molecular Modeling and Dynamics Studies of Proteins. *J Phys Chem B* **1998**, *102* (18), 3586–3616. https://doi.org/10.1021/jp973084f.

(4) Phillips, J. C.; Hardy, D. J.; Maia, J. D. C.; Stone, J. E.; Ribeiro, J. v.; Bernardi, R. C.; Buch, R.; Fiorin, G.; Hénin, J.; Jiang, W.; McGreevy, R.; Melo, M. C. R.; Radak, B. K.; Skeel, R. D.; Singharoy, A.; Wang, Y.; Roux, B.; Aksimentiev, A.; Luthey-Schulten, Z.; Kalé, L. v.; Schulten, K.; Chipot, C.; Tajkhorshid, E. Scalable Molecular Dynamics on CPU and GPU Architectures with NAMD. *Journal of Chemical Physics* **2020**, *153* (4), 044130. https://doi.org/10.1063/5.0014475.

(5) Jorgensen, W. L.; Chandrasekhar, J.; Madura, J. D.; Impey, R. W.; Klein, M. L. Comparison of Simple Potential Functions for Simulating Liquid Water. *J Chem Phys* **1983**, *79* (2), 926–935. https://doi.org/10.1063/1.445869.

(6) Darden, T.; York, D.; Pedersen, L. Particle Mesh Ewald: An N·log(N) Method for Ewald Sums in Large Systems. *J Chem Phys* **1993**, *98* (12), 10089–10092. https://doi.org/10.1063/1.464397.

(7) Ryckaert, J.-P.; Ciccotti, G.; Berendsen, H. J. C. Numerical Integration of the Cartesian Equations of Motion of a System with Constraints: Molecular Dynamics of n-Alkanes. *J Comput Phys* **1977**, *23* (3), 327–341. https://doi.org/10.1016/0021-9991(77)90098-5.

(8) Becke, A. D. Density-functional Thermochemistry. III. The Role of Exact Exchange. *J Chem Phys* **1993**, *98* (7), 5648–5652. https://doi.org/10.1063/1.464913.

(9) Ditchfield, R.; Hehre, W. J.; Pople, J. A. Self-Consistent Molecular-Orbital Methods. IX. An Extended Gaussian-Type Basis for Molecular-Orbital Studies of Organic Molecules. *J Chem Phys* **1971**, *54* (2), 724–728. https://doi.org/10.1063/1.1674902.

(10) Hehre, W. J.; Ditchfield, R.; Pople, J. A. Self—Consistent Molecular Orbital Methods. XII. Further Extensions of Gaussian—Type Basis Sets for Use in Molecular Orbital Studies of Organic Molecules. *J Chem Phys* **1972**, *56* (5), 2257–2261. https://doi.org/10.1063/1.1677527.

(11) Mardirossian, N.; Head-Gordon, M. ω-B97M-V: A Combinatorially Optimized, Range-Separated Hybrid, Meta-GGA Density Functional with VV10 Nonlocal Correlation. *J Chem Phys* **2016**, *144* (21). https://doi.org/10.1063/1.4952647.

(12) Dunning, T. H. Gaussian Basis Sets for Use in Correlated Molecular Calculations. I. The Atoms Boron through Neon and Hydrogen. *J Chem Phys* **1989**, *90* (2), 1007–1023. https://doi.org/10.1063/1.456153.

(13) Rosta, E.; Woodcock, H. L.; Brooks, B. R.; Hummer, G. Artificial Reaction Coordinate "Tunneling" in Free-Energy Calculations: The Catalytic Reaction of RNase H. *J Comput Chem* **2009**, *30* (11), 1634–1641. https://doi.org/10.1002/jcc.21312.

(14) Shao, Y.; Gan, Z.; Epifanovsky, E.; Gilbert, A. T. B.; Wormit, M.; Kussmann, J.; Lange, A. W.; Behn, A.; Deng, J.; Feng, X.; Ghosh, D.; Goldey, M.; Horn, P. R.; Jacobson, L. D.; Kaliman, I.; Khaliullin, R. Z.; Kuś, T.; Landau, A.; Liu, J.; Proynov, E. I.; Rhee, Y. M.; Richard, R. M.; Rohrdanz, M. A.; Steele, R. P.; Sundstrom, E. J.; Woodcock, H. L.; Zimmerman, P. M.; Zuev, D.; Albrecht, B.; Alguire, E.; Austin, B.; Beran, G. J. O.; Bernard, Y. A.; Berquist, E.; Brandhorst, K.; Bravaya, K. B.; Brown, S. T.; Casanova, D.; Chang, C.-M.; Chen, Y.; Chien, S. H.; Closser, K. D.; Crittenden, D. L.; Diedenhofen, M.; DiStasio, R. A.; Do, H.; Dutoi, A. D.; Edgar, R. G.; Fatehi, S.; Fusti-Molnar, L.; Ghysels, A.; Golubeva-Zadorozhnaya, A.; Gomes, J.; Hanson-Heine, M. W. D.; Harbach, P. H. P.; Hauser, A. W.; Hohenstein, E. G.; Holden, Z. C.; Jagau, T.-C.; Ji, H.; Kaduk, B.; Khistyaev, K.; Kim, J.; Kim, J.; King, R. A.; Klunzinger, P.; Kosenkov, D.; Kowalczyk, T.; Krauter, C. M.; Lao, K. U.; Laurent, A. D.; Lawler, K. v.; Levchenko, S. v.; Lin, C. Y.; Liu, F.; Livshits, E.; Lochan, R. C.; Luenser, A.; Manohar, P.; Manzer, S. F.; Mao, S.-P.; Mardirossian, N.; Marenich, A. v.; Maurer, S. A.; Mayhall, N. J.; Neuscamman, E.; Oana, C. M.; Olivares-Amaya, R.; O'Neill, D. P.; Parkhill, J. A.; Perrine, T. M.; Peverati, R.; Prociuk, A.; Rehn, D. R.; Rosta, E.; Russ, N. J.; Sharada, S. M.; Sharma, S.; Small, D. W.; Sodt, A.; Stein, T.; Stück, D.; Su, Y.-C.; Thom, A. J. W.; Tsuchimochi, T.; Vanovschi, V.; Vogt, L.; Vydrov, O.; Wang, T.; Watson, M. A.; Wenzel, J.; White, A.; Williams, C. F.; Yang, J.; Yeganeh, S.; Yost, S. R.; You, Z.-Q.; Zhang, I. Y.; Zhang, X.; Zhao, Y.; Brooks, B. R.; Chan, G. K. L.; Chipman, D. M.; Cramer, C. J.; Goddard, W. A.; Gordon, M. S.; Hehre, W. J.; Klamt, A.; Schaefer, H. F.; Schmidt, M. W.; Sherrill, C. D.; Truhlar, D. G.; Warshel, A.; Xu, X.; Aspuru-Guzik, A.; Baer, R.; Bell, A. T.; Besley, N. A.; Chai, J.-D.; Dreuw, A.; Dunietz, B. D.; Furlani, T. R.; Gwaltney, S. R.; Hsu, C.-P.; Jung, Y.; Kong, J.; Lambrecht, D. S.; Liang, W.; Ochsenfeld, C.; Rassolov, V. A.; Slipchenko, L. v.; Subotnik, J. E.; van Voorhis, T.; Herbert, J. M.; Krylov, A. I.; Gill, P. M. W.; Head-Gordon, M. Advances in Molecular Quantum Chemistry Contained in the Q-Chem 4 Program Package. *Mol Phys* **2015**, *113* (2), 184–215. https://doi.org/10.1080/00268976.2014.952696.

(15) Woodcock, H. L.; Hodošček, M.; Gilbert, A. T. B.; Gill, P. M. W.; Schaefer, H. F.; Brooks, B. R. Interfacing Q-Chem and CHARMM to Perform QM/MM Reaction Path Calculations. *J Comput Chem* **2007**, *28* (9), 1485–1502. https://doi.org/10.1002/JCC.20587.

(16) E, W.; Ren, W.; Vanden-Eijnden, E. Finite Temperature String Method for the Study of Rare Events. *J Phys Chem B* **2005**, *109* (14), 6688–6693. https://doi.org/10.1021/jp0455430.

(17) Rosta, E.; Nowotny, M.; Yang, W.; Hummer, G. Catalytic Mechanism of RNA Backbone Cleavage by Ribonuclease H from Quantum Mechanics/Molecular Mechanics Simulations. *J Am Chem Soc* **2011**, *133* (23), 8934–8941. https://doi.org/10.1021/ja200173a.

(18) Kumar, S.; Rosenberg, J. M.; Bouzida, D.; Swendsen, R. H.; Kollman, P. A. The Weighted Histogram Analysis Method for Free-Energy Calculations on Biomolecules. I. The Method. *J Comput Chem* **1992**, *13* (8), 1011–1021. https://doi.org/10.1002/jcc.540130812.

(19) Reed, A. E.; Weinstock, R. B.; Weinhold, F. Natural Population Analysis. *J Chem Phys* **1985**, *83* (2), 735–746. https://doi.org/10.1063/1.449486.

(20) Frisch, M. J.; Trucks, G. W.; Schlegel, H. B.; Scuseria, G. E.; Robb, M. A.; Cheeseman, J. R.; Scalmani, G.; Barone, V.; Mennucci, B.; Petersson, G. A.; Nakatsuji, H.; Caricato, M.; Li, X.; Hratchian, H. P.; Izmaylov, A. F.; Bloino, J.; Zheng, G.; Sonnenberg, J. L.; Hada, M.; Ehara, M.; Toyota, K.; Fukuda, R.; Hasegawa, J.; Ishida, M.; Nakajima, T.; Honda, Y.; Kitao, O.; Nakai, H.; Vreven, T.; Montgomery Jr., J. A.; Peralta, J. E.; Ogliaro, F.; Bearpark, M.; Heyd, J. J.; Brothers, E.; Kudin, K. N.; Staroverov, V. N.; Kobayashi, R.; Normand, J.; Raghavachari, K.; Rendell, A.; Burant, J. C.; Iyengar, S. S.; Tomasi, J.; Cossi, M.; Rega, N.; Millam, J. M.; Klene, M.; Knox, J. E.; Cross, J. B.; Bakken, V.; Adamo, C.; Jaramillo, J.; Gomperts, R.; Stratmann, R. E.; Yazyev, O.; Austin, A. J.; Cammi, R.; Pomelli, C.; Ochterski, J. W.; Martin, R. L.; Morokuma, K.; Zakrzewski, V. G.; Voth, G. A.; Salvador, P.; Dannenberg, J. J.; Dapprich, S.; Daniels, A. D.; Farkas, Ö.; Foresman, J. B.; Ortiz, J. v; Cioslowski, J.; Fox, D. J. Gaussian09 Revision E.01.
